# Sprinting to the top: comparing quality of distance variety and specialization between swimmers and runners

**DOI:** 10.3389/fspor.2024.1431594

**Published:** 2024-08-05

**Authors:** Dennis-Peter Born, Michael Romann, Jenny Lorentzen, David Zumbach, Andri Feldmann, Jesús J. Ruiz-Navarro

**Affiliations:** ^1^ Swiss Development Hub for Strength and Conditioning in Swimming, Swiss Swimming Federation, Worblaufen, Switzerland; ^2^ Department for Elite Sport, Swiss Federal Institute of Sport Magglingen, Magglingen, Switzerland; ^3^ Faculty of Science and Medicine, University of Fribourg, Fribourg, Switzerland; ^4^ Performance Sport, Swiss Athletics Federation, Ittigen, Switzerland; ^5^ Institute of Sport Science, University of Bern, Bern, Switzerland; ^6^ Aquatics Lab, Department of Physical Education and Sports, Faculty of Sport Sciences, University of Granada, Granada, Spain

**Keywords:** competition, elite athlete, talent, competitive swimming, diversification, long-term athlete development, sampling

## Abstract

**Objectives:**

To compare performance progression and variety in race distances of comparable lengths (timewise) between pool swimming and track running. Quality of within-sport variety was determined as the performance differences between individual athletes' main and secondary race distances across (top-) elite and (highly-) trained swimmers and runners.

**Methods:**

A total of 3,827,947 race times were used to calculate performance points (race times relative to the world record) for freestyle swimmers (*n* = 12,588 males and *n* = 7,561 females) and track runners (*n* = 9,230 males and *n* = 5,841 females). Athletes were ranked based on their personal best at peak performance age, then annual best times were retrospectively traced throughout adolescence.

**Results:**

Performance of world-class swimmers differentiates at an earlier age from their lower ranked peers (15–16 vs. 17–20 year age categories, *P* < 0.05), but also plateaus earlier towards senior age compared to runners (19–20 vs. 23 + year age category, *P* < 0.05), respectively. Performance development of swimmers shows a logarithmic pattern, while runners develop linearly. While swimmers compete in more secondary race distances (larger within-sport variety), runners specialize in either sprint, middle- or long-distance early in their career and compete in only 2, 4 or 3 other race distances, respectively. In both sports, sprinters specialize the most (*P* < 0.05). Distance-variety of middle-distance swimmers covers more longer rather than sprint race distances. Therefore, at peak performance age, (top-) elite female 200 m swimmers show significantly slower sprint performances, i.e., 50 m (*P* < 0.001) and 100 m (*P* < 0.001), but not long-distance performances, i.e., 800 m (*P* = 0.99) and 1,500 m (*P* = 0.99). In contrast, (top-) elite female 800 m middle-distance runners show significantly slower performances in all their secondary race distances (*P* < 0.001). (Top-) elite female athletes specialize more than (highly-) trained athletes in both sports (*P* < 0.05).

**Conclusions:**

The comparison to track running and lower ranked swimmers, the early performance plateau towards senior age, and the maintenance of a large within-sport distance variety indicates that (top-) elite sprint swimmers benefit from greater within-sport specialization.

## Introduction

Specializing late and maintaining a large variety of sport disciplines supposedly increase the chances of top performances at elite age, limit burnout risk and reduce injury incidences (1–3). However, evidence is limited to review articles and only a low number of original investigations (4). Additionally, many findings originate from game sports (5), which profit from skill transfer between disciplines, i.e., tactical positioning and decision-making (6), and limit their implications for cgs- (centimeter-gram-second) and individual sports. Furthermore, accuracy of retrospective questionnaires on sport participation during junior age are limited to the participants' accurate memory and only assess quantity of sport variety (3, 7, 8). Assessment of the quality of within-sport variety, i.e., performance differences between the athletes' main and secondary events, may further improve knowledge of specialization and variety during long-term athlete development (LTAD).

The quality of within-sport variety can be assessed by comparing performance over various race distances. Swimming conditions are standardized during competitions, i.e., specified water temperature, limited current, exact pool length and wave breaking lane ropes (9). This allows the comparison of race results from various venues and championships to analyze variety in race distances and throughout an athlete's entire swimming career. As such, previous studies found that swimmers typically enter the sport early and, on average, accumulate 8 years of competition participation before reaching (top-) elite level (10, 11), but maintain a larger skill variety within their sport (12). Competing in more than one swimming stroke and race distance as a child may indeed improve success and medal chances at adult age, particularly for freestyle events, which provide up to six race distances from 50 m to 1,500 m (13, 14).

However, large within-sport variety may also limit performance progression, as the ever-evolving landscape of swimming and continuously improving world records (15–17) require new training and development strategies to meet the distance-specific biomechanical and physiological requirements (18, 19). Sprint swimmers in particular may benefit a more intense, race-pace specific, and less voluminous training approach to optimally transfer high stroke frequencies into propulsion, develop neuro-muscular abilities, explosivity, speed and power for start and turn performances (20–23). Compared to the over-distance oriented approach in swimming, track runners typically follow training regimes that are characterized by more under-distance training: fewer hours per week with higher training intensities (24–27). This approach may be used to reduce overuse injuries, e.g., fatigue fractures, which are associated with impact forces and high volumes of running (28). Furthermore, the majority of disciplines in track-and-field involve explosive movement patterns, which typically require under-distance oriented training regimes with higher exercise intensities (25, 27, 29).

Additionally, track runners do not maintain the same within-sport variety as swimmers, but instead specialize in either sprint-, middle- and long-distance races (12, 27, 30). Despite their very different approaches to training, pool swimming and track running share common race lengths in competition (timewise, Table 1) as well as common physiological and metabolic demands for sprint, middle- and long-distance events (33–37). Therefore, comparing LTAD and evolution of within-sport distance variety between track runners and pool swimmers may provide new insights into training and development strategies, particularly for the neuro-muscular adaptations of sprint swimmers.

**Table 1:** World record times [mm:ss.00] of long-course (50 m pool length) freestyle swimming and outdoor (400 m track length) track running events (31, 32).

Pool swimming			Track running		
	Women	Men		Women	Men
Sprint					
50 m	00:23.61	00:20.91	200 m	00:21.34	00:19.19
100 m	00:51.71	00:46.80	400 m	00:47.60	00:43.03
Middle-distance					
200 m	01:52.85	01:42.00	800 m	01:53.28	01:40.91
400 m	03:55.38	03:40.07	1,500 m	03:49.11	03:26.00
Long-distance					
800 m	08:04.79	07:32.12	3,000 m	08:06.11	07:20.67
1,500 m	15:20.48	14:31.02	5,000 m	14:00.21	12:35.36

The aim of the present study was to compare performance progression and variety in race distances of comparable lengths (timewise) between pool swimming and track running. Quality of within-sport variety was assessed between the athletes' main and secondary race distances as well as between various performance levels. Track runners were expected to show lower variety and higher specialization on and around their main race distances.

## Methods

### Subjects

A total of 3,827,947 race times of freestyle pool swimmers (*n* = 12,588 males and *n* = 7,561 females) and track runners (*n* = 9,230 males and *n* = 5,841 females) representing 213 countries were obtained from the official databases of the European Aquatics (38) and World Athletics associations (39) and used for the present study. The data set of the male swimmers has previously been used as part of another study (12), however, to provide a comprehensive data set and comparison to the male runners' race data, two additional years (2022 and 2023) and another performance level (regional-class swimmers) have been added since the mentioned publication. Additionally, we have further developed the statistical model from repeated measure analysis of variance (ANOVA) to linear mixed model analysis (LMM). As only publicly available data are included and were analyzed anonymously, explicit written consent from the athletes was not required. The study protocol received prior approval from the institutional review board of the Swiss Federal Institute of Sport Magglingen (Reg.-Nr. 222_LSP_Born_03_2024) and adheres to the ethical standards outlined in the Declaration of Helsinki by the World Medical Association regarding research with human subjects.

### Data collection

To compare performance progression and variety in race distances of comparable lengths (timewise, refer to Table 1) and to determine factors leading to world-class performances at peak performance age in swimming and track running regarding performance progression and quality of within-sport distance variety, only swimmers and runners still competing at peak performance age were included in the present study. Hence, all swimmers and track runners at peak performance age were extracted from the 2023 to 2016 databases and ranked based on their personal best in each particular race distance at peak performance age. Then, each individual athlete's annual best times for all race distances were retrospectively extracted until early junior age (13–14 year age category), i.e., also including the 2015–2006 databases.

Although, individual athletes may perform at the highest international level at a younger age, previous literature has shown that, on average, swimmers and track runners reach their peak performance aged 23–30 years (40, 41). Additionally, the recently introduced U23 European swimming championships, which is intended as a transition phase between international junior and senior championships (42), further supports the 23 year cut-off age that distinguishes between developing and peak performing swimmers and runners. Consistent with the Olympic events, which high-performance swimmers and runners aim for, only long-course swimming races (50 m pool length) and outdoor running races (400 m track) were considered for the present study. Freestyle provides the largest range in swimming race distances at Olympic swimming competitions (50 m to 1,500 m) and, therefore, provides a reasonable data base for the comparison with track running. Hence, only freestyle swimming races are included in the present study.

### Data analysis

All race times were converted to performance points according to the official method of the world governing body in swimming. The point system expresses race times relative to the current world record (which equals 1,000 points) and allows the comparison of performances across various race distances (9), and thus, the comparison between sports. With swimming as the main point of interest for the present study, the swimming-specific point system was used for both sports to cluster swimmers and runners into different performance levels. Those performance levels were based on previous recommendations (43) and each individual athlete's personal best at peak performance age: world-class finalists (>850 performance points), international-class (850-750 performance points), national-class (750-650 performance points) and regional-class (650-550 performance points). Athletes with fewer than 550 performance points were excluded from the data analysis.

Quality of within-sport variety was then compared across six race distances (refer to Table 1) for both swimmers and runners. Performances of the athletes' main race distance (the particular race distance on which the ranking at peak performance age was based) were compared with their individual performances in the other (secondary) race distances. For the sake of clarity and to make the data tsunami of the present study more accessible for the reader, only two performance groups were used to assess the quality of variety and comparison between the athletes' main and secondary race distances: (top-) elite (1,000-750) and (highly-) trained (750-550 performance points) swimmers and runners (44). To assess longitudinal development, annual best times were averaged over two years and compared across the 13–14, 15–16, 17–18, 19–20, 21–22 and 23–30 year age categories.

The “pandas” library (version 1.5.1, pandas-dev/pandas, Zenodo, Genève, Switzerland) in Python (version 3.9.7, Python Software Foundation, Beaverton, USA) was used to compute performance points, establish rankings at peak performance age, and extract annual best times retrospectively for all included swimmers and runners. The subsequent data processing was carried out with Microsoft Excel (version 2209, Microsoft Corporation, Redmond, WA, USA). As part of the present interdisciplinary study, the data collection and analysis were led by an experienced data scientist, holding a master's degree and PhD. Scripts and procedures were validated by the other scientists involved in the project.

### Statistical analysis

All data are presented as mean ± standard deviation and were analyzed with the Jamovi software package version 2.3.28.0 (Jamovi Project 2022, retrieved from https://www.jamovi.org). A diagonal straight line in the Q-Q plot and a Gaussian distribution in the histogram confirmed normally distributed standardized residuals (45). Non-normally distributed data were subjected to logarithmic transformation. Linear mixed model (LMM) analysis was used to compare performance levels (850 vs. 750 vs. 650 vs. 550 or international vs. national) across the age categories (13–14 vs. 15–16 vs. 17–18 vs. 19–20 vs. 21–22 vs. 23+) as fixed factors and athletes' performance points for the particular age category as dependent variable. Subject was added as the random factor to account for missing values in the time series. Fixed intercepts and restricted maximum likelihood (REML) were employed. Bonferroni's correction was used to correct *post hoc* tests for multiple pairwise comparisons. An alpha error of 0.05 determined significant differences. Since maturational growth rates are different between sexes, all analyses were conducted separately for men and women (46).

## Results

### Performance progression

**Figure 1:**
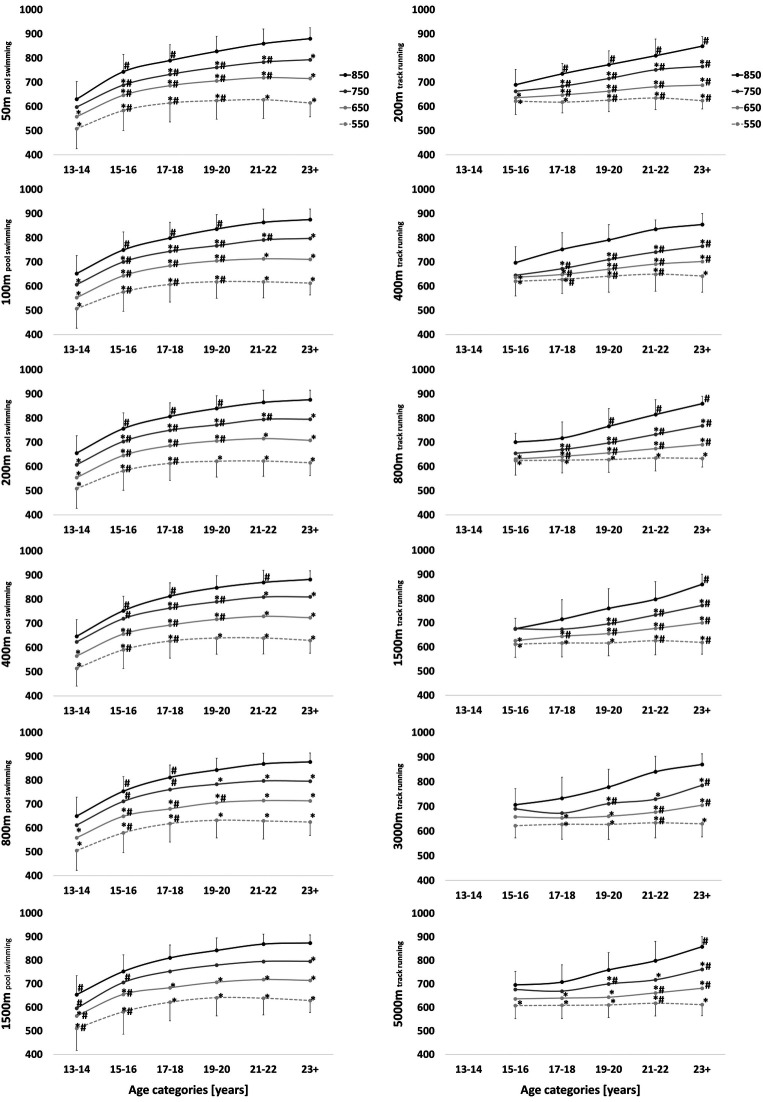
Progression of performance points [a.u.] over the age categories [years] of world-class finalists (850), international- (750), national- (650) and regional-class (550) ***female*** *pool **swimmers** and track **runners***. Athletes were ranked based on their personal best at peak performance age in the particular race distance. Annual best times across the age categories were retrospectively extracted across all race distances and compared between performance levels and age categories with linear mixed model analysis. Significant differences are indicated compared to world-class finalists (*) and the previous age category (#).

Performance progression of pool swimmers shows a logarithmic pattern: a steeper incline during the younger age categories and a flattening off towards peak performance age. In contrast, track runners show a linear development pattern (Figure 1). The earlier performance plateau in swimmers occurs between 17 and 22 years of age, while runners typically progress until the 23 + age category (*P* < 0.05, refer to Figure 1 and Table 2). While performance plateaus earlier in long-distance compared to sprint- and middle-distance swimmers, this is not the case for runners. However, performance of both female regional-class swimmers and runners declined towards the 23 + age category. In male regional-class athletes, this performance decline towards the 23 + age category is particularly evident in sprint swimmers, i.e., 50 m (*P* < 0.001) and 100 m (*P* = 0.007), as well as sprint, middle- and long-distance runners, i.e., 200 m (*P* < 0.001), 400 m (*P* = 0.012), 1,500 m (*P* < 0.001) and 5,000 m (*P* < 0.001). Furthermore, female swimmers' performances differentiate between the performance levels at a younger age compared to female track runners. Specifically, female world-class finalists start showing significantly faster race times compared to international-class athletes at a younger age in swimming compared to running (*P* < 0.05). Similar differences in differentiation between the two sports can also be seen in male athletes (Table 2).

**Table 2:** Progression of performance points [a.u.] over the various age categories [years] of world-class finalists (850), international- (750), national- (650) and regional-class (550) ***male*** *pool **swimmers** and track **runners***.

Performance level		Age categories [years]						Linear mixed model analysis		
		13–14	15–16	17–18	19–20	21–22	23+			
Pool swimmers										
50 m	850	512 ± 107	643 ± 92^#^	753 ± 81^#^	821 ± 65^#^	871 ± 54^#^	881 ± 34	*R*^2^_c_ = 0.83 ICC = 0.54	(a) *F*_[5|13965]_ = 1,949 (b) *F*_[3|4203]_ = 650 (c) *F*_[15|14010]_ = 31	*P* < 0.001 *P* < 0.001 *P* < 0.001
	750	472 ± 78	632 ± 82^#^	727 ± 73^#^	773 ± 66^#^	802 ± 57*^,#^	810 ± 46*			
	650	452 ± 85*	591 ± 81*^,#^	674 ± 74*^,#^	708 ± 69*^,#^	724 ± 73*^,#^	721 ± 56*			
	550	415 ± 87*	542 ± 88*^,#^	613 ± 81*^,#^	637 ± 80*^,#^	646 ± 78*^,#^	627 ± 62*^,#^			
100 m	850	510 ± 78	667 ± 78^#^	768 ± 69^#^	821 ± 57^#^	858 ± 50^#^	869 ± 33	*R*^2^_c_ = 0.85 ICC = 0.48	(a) *F*_[5|15667]_ = 6,037 (b) *F*_[3|4530]_ = 1,520 (c) *F*_[15|15721]_ = 27	*P* < 0.001 *P* < 0.001 *P* < 0.001
	750	478 ± 84*	627 ± 80*^,#^	717 ± 66*^,#^	760 ± 60*^,#^	781 ± 55*^,#^	785 ± 41*			
	650	443 ± 83*	584 ± 82*^,#^	661 ± 75*^,#^	691 ± 69*^,#^	704 ± 65*^,#^	699 ± 50*			
	550	400 ± 80*	520 ± 84*^,#^	589 ± 76*^,#^	611 ± 72*^,#^	616 ± 70*	603 ± 49*^,#^			
200 m	850	534 ± 89	697 ± 76^#^	795 ± 68^#^	846 ± 60^#^	879 ± 47	882 ± 34	*R*^2^_c_ = 0.84 ICC = 0.45	(a) *F*_[5|9303]_ = 3,366 (b) *F*_[3|2448]_ = 803 (c) *F*_[15|9324]_ = 10	*P* < 0.001 *P* < 0.001 *P* < 0.001
	750	493 ± 79*	659 ± 75*^,#^	754 ± 63*^,#^	796 ± 55*^,#^	816 ± 55*^,#^	811 ± 50*			
	650	471 ± 85*	616 ± 78*^,#^	695 ± 70*^,#^	727 ± 64*^,#^	740 ± 63*^,#^	732 ± 55*			
	550	423 ± 83*	550 ± 81*^,#^	623 ± 71*^,#^	646 ± 69*^,#^	655 ± 69*	643 ± 60*			
400 m	850	522 ± 88	701 ± 66^#^	800 ± 59^#^	852 ± 53^#^	882 ± 45	879 ± 40	*R*^2^_c_ = 0.85 ICC = 0.48	(a) *F*_[5|5591]_ = 3,358 (b) *F*_[3|1434]_ = 568 (c) *F*_[15|5599]_ = 9	*P* < 0.001 *P* < 0.001 *P* < 0.001
	750	493 ± 89	662 ± 82*^,#^	753 ± 67*^,#^	792 ± 59*^,#^	806 ± 52*	797 ± 46*			
	650	453 ± 80*	604 ± 77*^,#^	682 ± 67*^,#^	712 ± 66*^,#^	723 ± 64*	712 ± 54*			
	550	428 ± 91*	545 ± 84*^,#^	615 ± 77*^,#^	632 ± 74*^,#^	640 ± 71*	625 ± 59*			
800 m	850	504 ± 87	708 ± 73^#^	817 ± 57^#^	860 ± 66	897 ± 47	902 ± 39	*R*^2^_c_ = 0.86 ICC = 0.51	(a) *F*_[5|2993]_ = 1,999 (b) *F*_[3|766]_ = 245 (c) *F*_[15|3001]_ = 8	*P* < 0.001 *P* < 0.001 *P* < 0.001
	750	511 ± 88	671 ± 82^#^	764 ± 67*^,#^	809 ± 56^#^	826 ± 52*	818 ± 44*			
	650	459 ± 80*	619 ± 76*^,#^	700 ± 69*^,#^	734 ± 68*^,#^	746 ± 65*	733 ± 54*			
	550	440 ± 89*	565 ± 87*^,#^	636 ± 85*^,#^	656 ± 83*^,#^	669 ± 74*	646 ± 60*			
1,500 m	850	502 ± 91	709 ± 77^#^	802 ± 69^#^	855 ± 59^#^	883 ± 50	883 ± 43	*R*^2^_c_ = 0.86 ICC = 0.50	(a) *F*_[5|2448]_ = 1,891 (b) *F*_[3|648]_ = 246 (c) *F*_[15|2450]_ = 8	*P* < 0.001 *P* < 0.001 *P* < 0.001
	750	486 ± 88	652 ± 87*^,#^	746 ± 66*^,#^	791 ± 57*^,#^	805 ± 51*	797 ± 41*			
	650	454 ± 74*	607 ± 74*^,#^	684 ± 71*^,#^	711 ± 67*^,#^	719 ± 64*	710 ± 50*			
	550	430 ± 88*	559 ± 81*^,#^	620 ± 78*^,#^	634 ± 79*	647 ± 76*	627 ± 56*			
Track runners										
200 m	850		687 ± 49	756 ± 47^#^	800 ± 44^#^	837 ± 48^#^	854 ± 39	*R*^2^_c_ = 0.75 ICC = 0.57	(a) *F*_[4|5680]_ = 352 (b) *F*_[3|5486]_ = 330 (c) *F*_[12|5767]_ = 74	*P* < 0.001 *P* < 0.001 *P* < 0.001
	750		690 ± 34	714 ± 44*^,#^	743 ± 49*^,#^	767 ± 51*^,#^	779 ± 40*^,#^			
	650		684 ± 38	694 ± 42*^,#^	705 ± 46*^,#^	714 ± 48*^,#^	707 ± 41*			
	550		668 ± 18	676 ± 33*	684 ± 36*	687 ± 40*	660 ± 32*^,#^			
400 m	850		698 ± 55	762 ± 51^#^	800 ± 56^#^	835 ± 53^#^	857 ± 42^#^	*R*^2^_c_ = 0.75 ICC = 0.60	(a) *F*_[4|5116]_ = 254 (b) *F*_[3|5030]_ = 213 (c) *F*_[12|5132]_ = 45	*P* < 0.001 *P* < 0.001 *P* < 0.001
	750		697 ± 36	721 ± 46*^,#^	750 ± 48*^,#^	768 ± 50*^,#^	777 ± 37*^,#^			
	650		680 ± 33	696 ± 41*^,#^	710 ± 47*^,#^	721 ± 51*^,#^	711 ± 47*^,#^			
	550		690 ± 43	698 ± 47*	698 ± 43*	704 ± 49*	680 ± 50*^,#^			
800 m	850		746 ± 52	770 ± 62^#^	811 ± 58^#^	845 ± 51^#^	863 ± 38^#^	*R*^2^_c_ = 0.73 ICC = 0.49	(a) *F*_[4|3987]_ = 466 (b) *F*_[3|4693]_ = 327 (c) *F*_[12|4027]_ = 58	*P* < 0.001 *P* < 0.001 *P* < 0.001
	750		714 ± 47	731 ± 44*^,#^	753 ± 47*^,#^	774 ± 46*^,#^	783 ± 38*^,#^			
	650		695 ± 27	709 ± 36*	720 ± 39*^,#^	727 ± 40*^,#^	719 ± 32*			
	550									
1,500 m	850		750 ± 47	758 ± 54	796 ± 60^#^	833 ± 53^#^	860 ± 37^#^	*R*^2^_c_ = 0.75 ICC = 0.58	(a) *F*_[4|4507]_ = 127 (b) *F*_[3|5717]_ = 230 (c) *F*_[12|4429]_ = 56	*P* < 0.001 *P* < 0.001 *P* < 0.001
	750		719 ± 51	721 ± 44*	744 ± 49*^,#^	765 ± 50*^,#^	780 ± 40*^,#^			
	650		709 ± 39*	706 ± 44*	713 ± 47*^,#^	721 ± 46*^,#^	715 ± 42*			
	550			693 ± 33*	703 ± 37*	704 ± 46*	660 ± 38*^,#^			
3,000 m	850			785 ± 87	808 ± 82^#^	838 ± 66^#^	866 ± 42^#^	*R*^2^_c_ = 0.79 ICC = 0.64	(a) *F*_[4|1642]_ = 103 (b) *F*_[3|2585]_ = 76 (c) *F*_[12|1653]_ = 18	*P* < 0.001 *P* < 0.001 *P* < 0.001
	750		723 ± 70	716 ± 51*	743 ± 60*^,#^	767 ± 55*^,#^	787 ± 42*^,#^			
	650		719 ± 74	706 ± 46*	707 ± 50*^,#^	713 ± 52*^,#^	712 ± 47*			
	550		679 ± 70	708 ± 52*	697 ± 46*	700 ± 48*	674 ± 42*			
5,000 m	850		792 ± 34	804 ± 72	821 ± 74^#^	840 ± 62	856 ± 44^#^	*R*^2^_c_ = 0.75 ICC = 0.58	(a) *F*_[4|2688]_ = 84 (b) *F*_[3|4215]_ = 166 (c) *F*_[12|2728]_ = 23	*P* < 0.001 *P* < 0.001 *P* < 0.001
	750		752 ± 52	734 ± 57*	743 ± 60*^,#^	760 ± 56*^,#^	780 ± 42*^,#^			
	650		688 ± 42*	695 ± 42*	706 ± 47*^,#^	715 ± 47*^,#^	709 ± 41*			
	550		689 ± 66*	698 ± 34*	694 ± 47*	694 ± 47*	667 ± 43*^,#^			

Athletes were ranked based on their personal best at peak performance age in the particular race distance. Annual best times across the age categories were retrospectively extracted across all race distances.

*R*^2^_c_, *R*-squared conditional; ICC, intra-class correlation coefficient.

Linear mixed model analysis:

a) Main effect: age category (13–14 vs. 15–16 vs. 17–18 vs. 19–20 vs. 21–22 vs. 23+).

b) Main effect: performance level (850 vs. 750 vs. 650 vs. 550).

c) Interaction effect: age category × performance level.

Bonferroni *post hoc* comparison:

* significant difference to the 850 performance level (world-class finalists).

^#^ significant difference to the previous age category.

### Quality of within-sport distance variety

**Figure 2:**
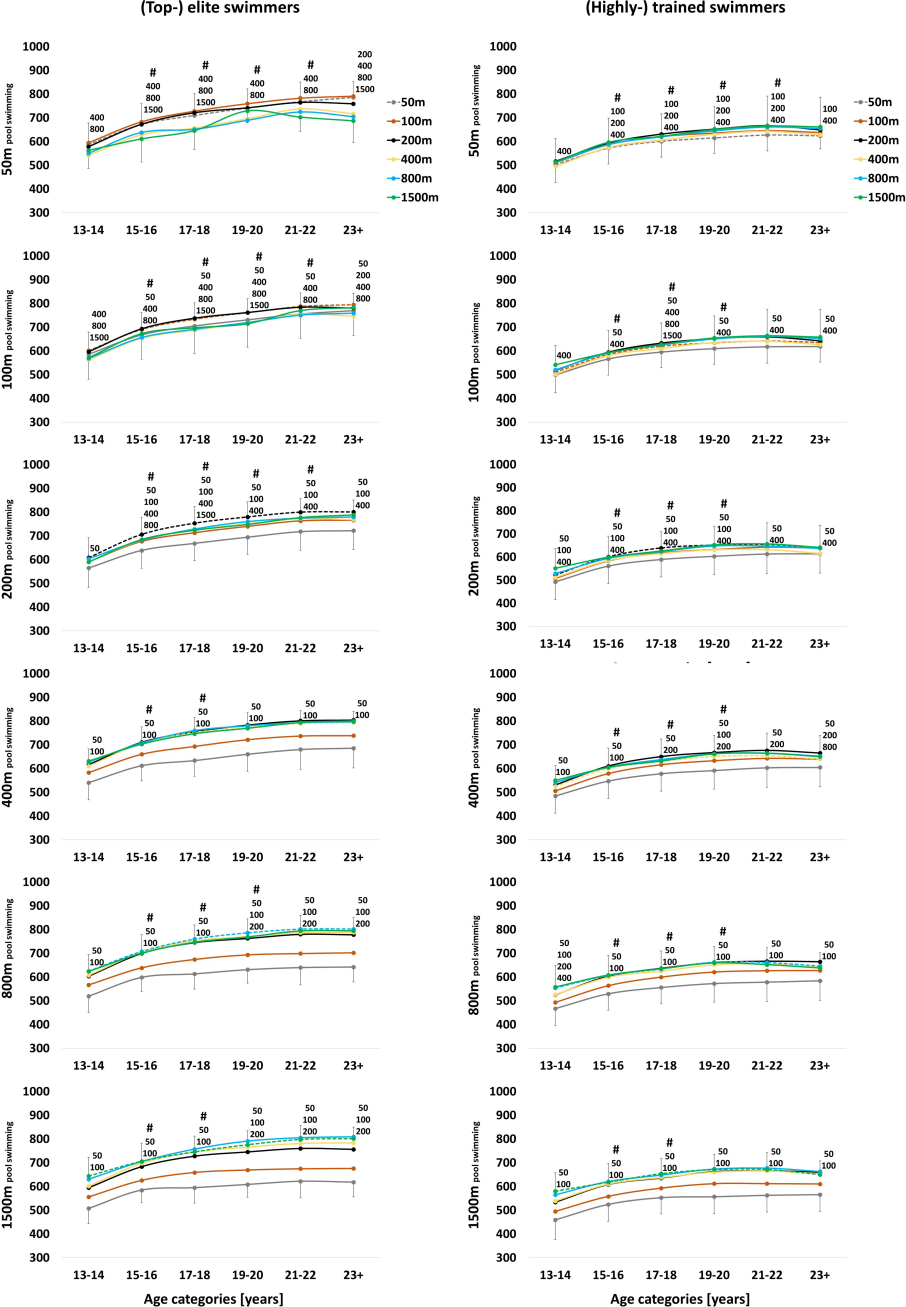
Progression of performance points [a.u.] of (top-) elite (1,000-750 performance points) and (highly-) trained ***female*** *freestyle pool **swimmers*** (750-550 performance points) across the various age categories [years]. Annual best times of the main race distance (dotted lines) were retrospectively extracted and compared to the individual swimmers’ annual best times of their secondary race distances using linear mixed model analysis, with ^50, 100, 200, 400, 800^ and ^1500^ indicating the significant difference to the specific secondary race distance. For the sake of clarity, significant differences to the previous age category (#) are only indicated for the main race distance.

**Figure 3:**
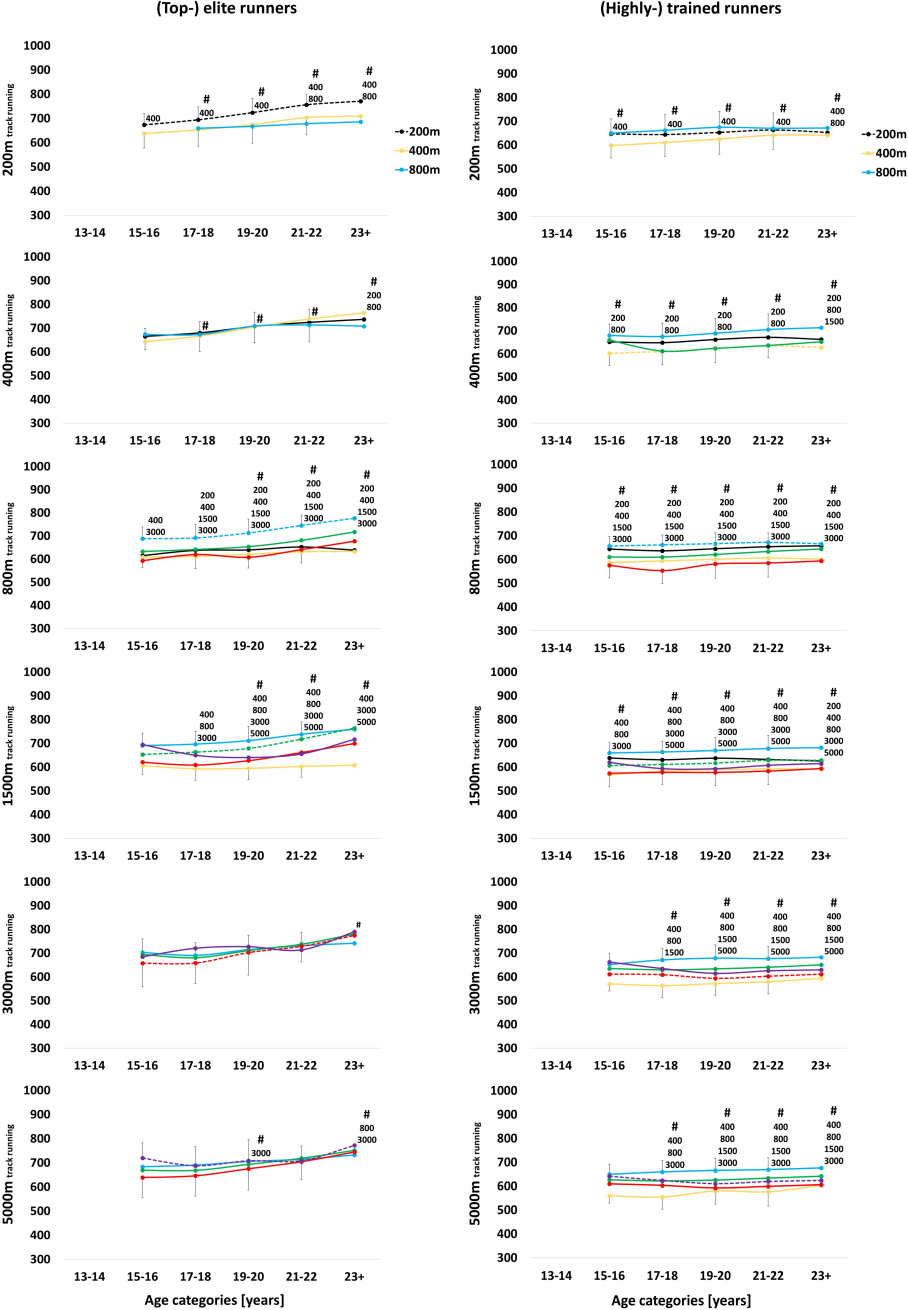
Progression of performance points [a.u.] of (top-) elite (1,000-750 performance points) and (highly-) trained ***female*** *track **runners*** (750-550 performance points) across the various age categories [years]. Annual best times of the main race distance (dotted lines) were retrospectively extracted and compared to the individual runners’ annual best times of their secondary race distances using linear mixed model analysis, with ^200, 400, 800, 1500, 3000^ and ^5000^ indicating significant differences to specific secondary race distances. For the sake of clarity, significant differences to the previous age category (#) are only indicated for the main race distance.

While swimmers typically compete across all race distances, runners specialize in either sprint, middle- or long-distance early in their career and compete in only two, four or three other race distances, respectively. In both sports, sprinters specialize more than middle- and long-distance athletes. As such, (top-) elite female 50 m swimmers show significantly slower performances in their 200 m (*P* < 0.001), 400 m (*P* < 0.001), 800 m (*P* < 0.001) and 1,500 m races (*P* = 0.029) at peak performance age, while (top-) elite female 200 m sprint runners do not even compete over race distances of more than 800 m. (Top-) elite female 800 m and 1,500 m swimmers show significantly slower performances in their 50 m (*P* < 0.001), 100 m (*P* < 0.001) and 200 m (*P* < 0.001) races at peak performance age. The variety of middle-distance swimmers covers more long- rather than sprint race distances. As such, at peak performance age, (top-) elite female 200 m swimmers show significantly slower sprint performances, i.e., 50 m (*P* < 0.001) and 100 m (*P* < 0.001), but not long-distance performances, i.e., 800 m (*P* = 0.99) and 1,500 m (*P* = 0.99) (23 + age category). Runners are more specialized, as (top-) elite female 800 m middle-distance runners show significantly slower performances in all their secondary race distances, i.e., 200 m, 400 m, 1,500 m, 3,000 m (all *P* < 0.001; Figures 2, 3).

(Top-) elite female athletes specialize more than (highly-) trained female athletes in both sports. While (top-) elite swimmers still compete across all six race distances at peak performance age (23 + age category), they show more significantly slower secondary race distances compared to their main race distance than (highly-) trained swimmers (*P* < 0.05). Specifically, (top-) elite female 100 m swimmers show significantly slower 50 m (*P* = 0.002), 200 m (*P* = 0.008), 400 m (*P* < 0.001) and 800 m (*P* < 0.001) race times, while (highly-) trained female 100 m swimmers only show significantly slower 50 m (*P* < 0.001) and 400 m (*P* < 0.001) race times at peak performance age. The same effect of greater specialization in higher ranked athletes is apparent in male swimmers. (Top-) elite female runners generally compete in a lower number of secondary race distances compared to (highly-) trained runners. For instance, (top-) elite female 400 m runners also compete in 200 m and 800 m races, while (highly-) trained 400 m runners compete in 200 m–1,500 m races. (Top-) elite female 5,000 m runners compete over all race distances from 800 m to 5,000 m, while (highly-) trained 5,000 m runners compete over all race distances from 400 m to 5,000 m. Male runners showed the opposite trend, with (top-) elite 800 m, 1,500 m and 5,000 m runners competing in more secondary race distances than (highly-) trained runners (Tables 3, 4).

**Table 3:** Progression of performance points [a.u.] of (top-) elite (1,000-750 performance points) and (highly-) trained male freestyle pool swimmers (750-550 performance points) across the various age categories [years].

Main and secondary race distances	Age categories [years]						Linear mixed model analysis		
	13–14	15–16	17–18	19–20	21–22	23+			
(Top-) elite pool swimmers									
**50 m**	**437 ± 80**	**590 ± 76^b^**	**682 ± 70^b^**	**730 ± 60^b^**	**765 ± 55^b^**	**781 ± 43**	$R_{c}^{2}$ = 0.79 ICC = 0.40	(a) *F*_[5|6763]_ = 1235 (b) *F*_[5|6756]_ = 243 (c) *F*_[25|6718]_ = 15	*P* < 0.001 *P* < 0.001 *P* < 0.001
100 m	468 ± 83^a^	630 ± 83^a^	725 ± 74^a^	777 ± 67^a^	804 ± 72^a^	810 ± 72^a^			
200 m	444 ± 82	589 ± 91	668 ± 89^a^	707 ± 90^a^	731 ± 96^a^	736 ± 96^a^			
400 m	458 ± 80	585 ± 95	652 ± 102^a^	699 ± 114^a^	719 ± 126^a^	732 ± 106^a^			
800 m	465 ± 69	546 ± 81^a^	611 ± 110^a^	649 ± 114^a^	687 ± 130^a^	681 ± 137^a^			
1,500 m	493 ± 67^a^	572 ± 89	631 ± 102^a^	659 ± 125^a^	650 ± 112^a^	646 ± 149^a^			
50 m	428 ± 77^a^	570 ± 73^a^	654 ± 69^a^	697 ± 65^a^	724 ± 65^a^	733 ± 60^a^	$R_{c}^{2}$ = 0.76 ICC = 0.38	(a) *F*_[5|16118]_ = 4369 (b) *F*_[5|16104]_ = 353 (c) *F*_[25|15991]_ = 18	*P* < 0.001 *P* < 0.001 *P* < 0.001
**100 m**	**468 ± 81**	**623 ± 78^b^**	**715 ± 67^b^**	**763 ± 59^b^**	**790 ± 60^b^**	**797 ± 48**			
200 m	457 ± 85^a^	600 ± 88^a^	686 ± 81^a^	727 ± 80^a^	747 ± 84^a^	746 ± 82^a^			
400 m	479 ± 88	613 ± 96	687 ± 102^a^	737 ± 100^a^	766 ± 103^a^	763 ± 98^a^			
800 m	472 ± 81	581 ± 86^a^	645 ± 99^a^	697 ± 99^a^	731 ± 97^a^	723 ± 107^a^			
1,500 m	509 ± 81^a^	612 ± 89^a^	674 ± 93^a^	732 ± 98^a^	737 ± 104^a^	728 ± 106^a^			
50 m	415 ± 74^a^	561 ± 71^a^	633 ± 73^a^	668 ± 71^a^	697 ± 73^a^	703 ± 73^a^	$R_{c}^{2}$ = 0.78 ICC = 0.33	(a) *F*_[5|11439]_ = 5210 (b) *F*_[5|11433]_ = 696 (c) *F*_[25|11359]_ = 7	*P* < 0.001 *P* < 0.001 *P* < 0.001
100 m	463 ± 77	624 ± 73	713 ± 68	758 ± 64	785 ± 69	789 ± 66			
**200 m**	**470 ± 79**	**629 ± 75^b^**	**722 ± 65^b^**	**766 ± 57^b^**	**791 ± 54^b^**	**791 ± 45**			
400 m	501 ± 85^a^	657 ± 83^a^	747 ± 77^a^	790 ± 69^a^	810 ± 75	800 ± 74			
800 m	491 ± 79^a^	621 ± 79	710 ± 76	746 ± 84	777 ± 80	773 ± 85			
1,500 m	524 ± 81^a^	657 ± 80^a^	730 ± 80	777 ± 87	799 ± 83	796 ± 88			
50 m	385 ± 73^a^	522 ± 75^a^	588 ± 72^a^	622 ± 68^a^	642 ± 76^a^	647 ± 78^a^	$R_{c}^{2}$ = 0.81 ICC = 0.39	(a) *F*_[5|11231]_ = 5716 (b) *F*_[5|11207]_ = 1574 (c) *F*_[25|11165]_ = 4	*P* < 0.001 *P* < 0.001 *P* < 0.001
100 m	441 ± 79^a^	591 ± 77^a^	671 ± 74^a^	713 ± 71^a^	734 ± 76^a^	735 ± 81^a^			
200 m	462 ± 80^a^	615 ± 75^a^	704 ± 67^a^	745 ± 64^a^	765 ± 67^a^	764 ± 65^a^			
**400 m**	**506 ± 88**	**661 ± 81^b^**	**755 ± 67^b^**	**795 ± 62^b^**	**812 ± 60**	**803 ± 54**			
800 m	496 ± 83	636 ± 79^a^	725 ± 71^a^	762 ± 69^a^	780 ± 65^a^	776 ± 69^a^			
1,500 m	531 ± 91*	671 ± 78	749 ± 75	793 ± 71	805 ± 70	801 ± 70			
50 m	366 ± 58^a^	495 ± 72^a^	559 ± 61^a^	594 ± 61^a^	610 ± 70^a^	607 ± 76^a^	$R_{c}^{2}$ = 0.86 ICC = 0.44	(a) *F*_[5|5925]_ = 3645 (b) *F*_[5|5912]_ = 1700 (c) *F*_[25|5901]_ = 2	*P* < 0.001 *P* < 0.001 *P* = 0.011
100 m	430 ± 72^a^	574 ± 74^a^	643 ± 74^a^	688 ± 65^a^	698 ± 73^a^	702 ± 78^a^			
200 m	461 ± 74^a^	607 ± 74^a^	691 ± 68^a^	729 ± 62^a^	749 ± 69^a^	748 ± 69^a^			
400 m	513 ± 78	667 ± 78	759 ± 66	802 ± 61	821 ± 60	813 ± 59			
**800 m**	**505 ± 73**	**649 ± 77^b^**	**741 ± 67^b^**	**782 ± 61^b^**	**800 ± 56**	**801 ± 49**			
1,500 m	542 ± 83^a^	684 ± 76^a^	766 ± 72^a^	808 ± 67^a^	824 ± 61^a^	821 ± 56^a^			
50 m	351 ± 55^a^	481 ± 75^a^	545 ± 62^a^	577 ± 61^a^	596 ± 72^a^	593 ± 75^a^	$R_{c}^{2}$ = 0.88 ICC = 0.48	(a) *F*_[5|5476]_ = 3609 (b) *F*_[5|5462]_ = 1787 (c) *F*_[25|5454]_ = 2	*P* < 0.001 *P* < 0.001 *P* = 0.017
100 m	409 ± 67^a^	556 ± 76^a^	626 ± 73^a^	670 ± 63^a^	678 ± 73^a^	676 ± 71^a^			
200 m	442 ± 72^a^	591 ± 77^a^	673 ± 67^a^	712 ± 61^a^	726 ± 68^a^	727 ± 67^a^			
400 m	498 ± 79^a^	654 ± 85^a^	748 ± 69	791 ± 64	809 ± 62	798 ± 62			
800 m	495 ± 72^a^	644 ± 77^a^	733 ± 70^a^	777 ± 63^a^	794 ± 59^a^	790 ± 55^a^			
**1,500 m**	**530 ± 87**	**679 ± 78^b^**	**761 ± 74^b^**	**807 ± 66^b^**	**823 ± 60**	**818 ± 53**			
(Highly-) trained pool swimmers									
**50 m**	**385 ± 76**	**504 ± 72^b^**	**577 ± 66^b^**	**608 ± 65^b^**	**625 ± 66^b^**	**626 ± 55**	$R_{c}^{2}$ = 0.73 ICC = 0.51	(a) *F*_[5|43650]_ = 8034 (b) *F*_[5|43614]_ = 462 (c) *F*_[25|43393]_ = 33	*P* < 0.001 *P* < 0.001 *P* < 0.001
100 m	412 ± 85^a^	546 ± 84^a^	625 ± 77^a^	658 ± 75^a^	675 ± 76^a^	667 ± 78^a^			
200 m	408 ± 87^a^	530 ± 89^a^	601 ± 92^a^	634 ± 95	649 ± 97	636 ± 102^a^			
400 m	431 ± 92^a^	549 ± 100^a^	615 ± 108^a^	648 ± 116	673 ± 117^a^	650 ± 128			
800 m	431 ± 84^a^	530 ± 90	593 ± 102	622 ± 111^a^	651 ± 115^a^	628 ± 132^a^			
1,500 m	465 ± 89^a^	560 ± 96^a^	624 ± 103^a^	654 ± 113	687 ± 107	650 ± 134			
50 m	371 ± 73^a^	486 ± 71^a^	553 ± 66^a^	585 ± 64^a^	601 ± 67^a^	599 ± 62^a^	$R_{c}^{2}$ = 0.75 ICC = 0.51	(a) *F*_[5|44467]_ = 11189 (b) *F*_[5|44381]_ = 620 (c) *F*_[25|44160]_ = 40	*P* < 0.001 *P* < 0.001 *P* < 0.001
**100 m**	**399 ± 81**	**528 ± 80^b^**	**605 ± 71^b^**	**635 ± 67^b^**	**647 ± 68^b^**	**638 ± 58**			
200 m	400 ± 82	520 ± 86^a^	590 ± 84^a^	617 ± 86^a^	627 ± 85^a^	608 ± 87^a^			
400 m	424 ± 86^a^	544 ± 96	610 ± 102	636 ± 109^a^	656 ± 110^a^	633 ± 119^a^			
800 m	429 ± 75^a^	533 ± 90^a^	596 ± 101^a^	627 ± 108^a^	651 ± 115^a^	622 ± 128^a^			
1,500 m	459 ± 82^a^	561 ± 95^a^	627 ± 101	657 ± 108	683 ± 110	648 ± 133^a^			
50 m	378 ± 74^a^	493 ± 72^a^	561 ± 71^a^	597 ± 71^a^	614 ± 78^a^	617 ± 78^a^	$R_{c}^{2}$ = 0.75 ICC = 0.49	(a) *F*_[5|30198]_ = 9397 (b) *F*_[5|30128]_ = 936 (c) *F*_[25|29984]_ = 33	*P* < 0.001 *P* < 0.001 *P* < 0.001
100 m	415 ± 82	546 ± 79	623 ± 72	657 ± 71	674 ± 76^a^	672 ± 73^a^			
**200 m**	**419 ± 82**	**548 ± 80^b^**	**621 ± 75^b^**	**651 ± 71^b^**	**659 ± 68**	**642 ± 59^b^**			
400 m	452 ± 88^a^	576 ± 87^a^	641 ± 88^a^	669 ± 89^a^	681 ± 86^a^	657 ± 88^a^			
800 m	450 ± 78^a^	557 ± 85	619 ± 91	654 ± 91	667 ± 94	646 ± 101			
1,500 m	481 ± 83^a^	586 ± 87^a^	648 ± 92^a^	682 ± 95^a^	696 ± 95^a^	678 ± 107^a^			
50 m	360 ± 71^a^	471 ± 74^a^	533 ± 74^a^	567 ± 74^a^	581 ± 80^a^	585 ± 78^a^	$R_{c}^{2}$ = 0.78 ICC = 0.54	(a) *F*_[5|16467]_ = 6032 (b) *F*_[5|16412]_ = 978 (c) *F*_[25|16370]_ = 25	*P* < 0.001 *P* < 0.001 *P* < 0.001
100 m	401 ± 80^a^	527 ± 79^a^	597 ± 77^a^	630 ± 77^a^	648 ± 83^a^	646 ± 81			
200 m	413 ± 78^a^	539 ± 79^a^	610 ± 75^a^	638 ± 75^a^	651 ± 74^a^	639 ± 71			
**400 m**	**449 ± 81**	**571 ± 84^b^**	**639 ± 83^b^**	**665 ± 78^b^**	**669 ± 78**	**643 ± 61^b^**			
800 m	448 ± 71	552 ± 79^a^	614 ± 84^a^	642 ± 81^a^	644 ± 81^a^	622 ± 72^a^			
1500 m	477 ± 76^a^	582 ± 80	640 ± 85	668 ± 82	670 ± 85	649 ± 80			
50 m	357 ± 75^a^	474 ± 80^a^	530 ± 79^a^	563 ± 76^a^	586 ± 80^a^	582 ± 80^a^	$R_{c}^{2}$ = 0.80 ICC = 0.54	(a) *F*_[5|9986]_ = 4146 (b) *F*_[5|9952]_ = 918 (c) *F*_[25S|9939]_ = 11	*P* < 0.001 *P* < 0.001 *P* < 0.001
100 m	400 ± 80^a^	528 ± 83^a^	593 ± 82^a^	628 ± 79^a^	653 ± 84^a^	644 ± 84			
200 m	417 ± 81^a^	549 ± 79^a^	618 ± 78^a^	646 ± 81	665 ± 79	652 ± 76			
400 m	453 ± 81	586 ± 82^a^	657 ± 85^a^	685 ± 83^a^	698 ± 78^a^	666 ± 73^a^			
**800 m**	**452 ± 73**	**570 ± 71**	**633 ± 80**	**661 ± 79**	**670 ± 73**	**644 ± 60**			
1,500 m	479 ± 81^a^	597 ± 75^a^	656 ± 82^a^	688 ± 79^a^	693 ± 75^a^	668 ± 69^a^			
50 m	353 ± 69^a^	468 ± 73^a^	520 ± 77^a^	550 ± 77^a^	565 ± 79^a^	563 ± 75^a^	$R_{c}^{2}$ = 0.80 ICC = 0.55	(a) *F*_[5|7478]_ = 2855 (b) *F*_[5|7451]_ = 701 (c) *F*_[25|7442]_ = 8	*P* < 0.001 *P* < 0.001 *P* < 0.001
100 m	396 ± 79^a^	523 ± 75^a^	584 ± 77^a^	614 ± 78^a^	631 ± 83^a^	624 ± 79^a^			
200 m	416 ± 76^a^	540 ± 75^a^	604 ± 77^a^	626 ± 80^a^	645 ± 81^a^	633 ± 75^a^			
400 m	447 ± 78^a^	578 ± 79	642 ± 82	666 ± 79	674 ± 79	650 ± 73			
800 m	449 ± 71^a^	564 ± 70^a^	622 ± 81	641 ± 79^a^	650 ± 72	626 ± 64^a^			
**1,500 m**	**477 ± 80**	**589 ± 74^b^**	**641 ± 81^b^**	**663 ± 78^b^**	**669 ± 74**	**647 ± 59^b^**			

Annual best times of the main race distance (dotted lines) were retrospectively extracted and compared to the individual swimmers’ annual best times of their secondary race distances. For the sake of clarity, significant differences to the previous age category are only indicated for the main race distance.

$R_{c}^{2}$, R-squared conditional; ICC, intra-class correlation coefficient.

Bold indicates main race distances.

Linear mixed model analysis: a) Main effect: age category (13–14 vs. 15–16 vs. 17–18 vs. 19–20 vs. 21–22 vs. 23+). b) Main effect: race distance (50 m vs. 100 m vs. 200 m vs. 400 m vs. 800 m vs. 1500 m). c) Interaction effect: age category × performance level.

Bonferroni *post-hoc* comparison:

^a^ Significant difference to the main race distance.

^b^ Significant difference to previous age category.

**Table 4:** Progression of performance points [a.u.] of (top-) elite (1,000-750 performance points) and (highly-) trained male track runners (750-550 performance points) across the various age categories [years].

Main and secondary race distances	Age categories [years]						Linear mixed model analysis		
	13–14	15–16	17–18	19–20	21–22	23+			
(Top-) elite track runners									
**200 m**		**686 ± 36**	**713 ± 47^b^**	**738 ± 50^b^**	**761 ± 52^b^**	**776 ± 41^b^**	$R_{c}^{2}$ = 0.51 ICC = 0.39	(a) *F*_[4|2660]_ = 11 (b) *F*_[2|2720]_ = 7 (c) *F*_[8|2636]_ = 6	*P* < 0.001 *P* = 0.001 *P* < 0.001
400 m		708 ± 43	730 ± 47	743 ± 60	772 ± 64	763 ± 67^a^			
800 m				710 ± 20	715 ± 40	722 ± 23			
200 m		671 ± 29^a^	696 ± 43^a^	716 ± 48^a^	728 ± 51^a^	730 ± 49^a^	$R_{c}^{2}$ = 0.62 ICC = 0.46	(a) *F*_[4|3337]_ = 127 (b) *F*_[2|3406]_ = 211 (c) *F*_[8|3300]_ = 2	*P* < 0.001 *P* < 0.001 *P* = 0.115
**400 m**		**705 ± 41**	**731 ± 49^b^**	**756 ± 52^b^**	**774 ± 54^b^**	**782 ± 43**			
800 m		707 ± 25	730 ± 49	752 ± 51	776 ± 60	776 ± 67			
200 m		653 ± 16^a^	671 ± 21^a^	679 ± 42^a^	673 ± 42^a^	687 ± 41^a^	$R_{c}^{2}$ = 0.67 ICC = 0.49	(a) *F*_[4|4699]_ = 79 (b) *F*_[4|4753]_ = 258 (c) *F*_[16|4669]_ = 6	*P* < 0.001 *P* < 0.001 *P* < 0.001
400 m		665 ± 28^a^	687 ± 38^a^	698 ± 43^a^	704 ± 49^a^	700 ± 48^a^			
**800 m**		**737 ± 39**	**743 ± 48**	**764 ± 49^b^**	**782 ± 50^b^**	**788 ± 45**			
1,500 m		740 ± 55	726 ± 44^a^	740 ± 52^a^	758 ± 57^a^	760 ± 60^a^			
3,000 m			666 ± 54^a^	691 ± 54^a^	702 ± 52^a^	725 ± 56^a^			
400 m		681 ± 33	678 ± 32^a^	686 ± 40^a^	686 ± 37^a^	680 ± 35^a^	$R_{c}^{2}$ = 0.65 ICC = 0.52	(a) *F*_[4|4372]_ = 111 (b) *F*_[3|4410]_ = 117 (c) *F*_[12|4335]_ = 12	*P* < 0.001 *P* < 0.001 *P* < 0.001
800 m		737 ± 43	734 ± 42	752 ± 49	767 ± 52	769 ± 53^a^			
**1,500 m**		**725 ± 55**	**727 ± 44**	**744 ± 49^b^**	**764 ± 52^b^**	**781 ± 44^b^**			
3,000 m		689 ± 29	670 ± 41^a^	696 ± 52^a^	716 ± 58^a^	741 ± 59^a^			
800 m			733 ± 33^a^	745 ± 44	757 ± 51	759 ± 45^a^	$R_{c}^{2}$ = 0.69 ICC = 0.62	(a) *F*_[4|1295]_ = 112 (b) *F*_[2|1273]_ = 10 (c) *F*_[8|1276]_ = 9	*P* < 0.001 *P* < 0.001 *P* < 0.001
1,500 m		770 ± 79	731 ± 49^a^	754 ± 55^a^	772 ± 58^a^	786 ± 52			
**3,000 m**		**732 ± 77**	**703 ± 71**	**729 ± 75^b^**	**749 ± 74^b^**	**781 ± 47^b^**			
800 m			742 ± 43	741 ± 47	760 ± 48	765 ± 51	$R_{c}^{2}$ = 0.65 ICC = 0.60	(a) *F*_[4|2017]_ = 99 (b) *F*_[3|2019]_ = 2 (c) *F*_[12|1994]_ = 3	*P* < 0.001 *P* = 0.081 *P* < 0.001
1,500 m		767 ± 36	747 ± 54	754 ± 57	770 ± 55	778 ± 54			
3,000 m		768 ± 63	718 ± 72	741 ± 82	758 ± 73	779 ± 56			
**5,000 m**		**771 ± 26**	**748 ± 75**	**737 ± 73**	**748 ± 65^b^**	**779 ± 46^b^**			
(Highly-) trained track runners									
**200 m**		**672 ± 33**	**678 ± 36^b^**	**686 ± 39^b^**	**692 ± 41^b^**	**681 ± 33^b^**	$R_{c}^{2}$ = 0.53 ICC = 0.45	(a) *F*_[4|6578]_ = 20 (b) *F*_[2|6746]_ = 152 (c) *F*_[8|6536]_ = 8	*P* < 0.001 *P* < 0.001 *P* < 0.001
400 m		686 ± 35	699 ± 43^a^	711 ± 46^a^	721 ± 49^a^	717 ± 47^a^			
800 m		731 ± 21	713 ± 22	721 ± 39^a^	731 ± 39^a^	736 ± 41^a^			
200 m		662 ± 33	676 ± 40^a^	684 ± 43^a^	690 ± 47^a^	684 ± 45^a^	$R_{c}^{2}$ = 0.59 ICC = 0.41	(a) *F*_[4|6171]_ = 102 (b) *F*_[2|6393]_ = 264 (c) *F*_[8|6059]_ = 23	*P* < 0.001 *P* < 0.001 *P* < 0.001
**400 m**		**676 ± 29**	**686 ± 37^b^**	**695 ± 37^b^**	**703 ± 40^b^**	**687 ± 31^b^**			
800 m		720 ± 23	730 ± 34^a^	749 ± 45^a^	762 ± 52^a^	765 ± 50^a^			
400 m		669 ± 26^a^	682 ± 45^a^	690 ± 45^a^	692 ± 51^a^	691 ± 46^a^	$R_{c}^{2}$ = 0.44 ICC = 0.37	(a) *F*_[4|3353]_ = 27 (b) *F*_[2|3393]_ = 68 (c) *F*_[8|3246]_ = 9	*P* < 0.001 *P* < 0.001 *P* < 0.001
**800 m**		**708 ± 19**	**715 ± 31**	**723 ± 36^b^**	**726 ± 35**	**712 ± 23^b^**			
1,500 m		682 ± 23	701 ± 31^a^	712 ± 32^a^	720 ± 38^a^	715 ± 42			
800 m		719 ± 21	719 ± 37^a^	729 ± 41^a^	735 ± 43^a^	735 ± 44^a^	$R_{c}^{2}$ = 0.54 ICC = 0.43	(a) *F*_[4|4572]_ = 46 (b) *F*_[2|4571]_ = 84 (c) *F*_[8|4368]_ = 18	*P* < 0.001 *P* < 0.001 *P* < 0.001
**1,500 m**		**732 ± 35**	**703 ± 37**	**708 ± 36^b^**	**715 ± 34^b^**	**694 ± 32^b^**			
3,000 m		659 ± 29	663 ± 32^a^	668 ± 38^a^	680 ± 44^a^	682 ± 43^a^			
800 m			718 ± 37^a^	723 ± 36^a^	733 ± 38^a^	727 ± 38^a^	$R_{c}^{2}$ = 0.59 ICC = 0.47	(a) *F*_[4|2344]_ = 31 (b) *F*_[2|2247]_ = 35 (c) *F*_[8|2260]_ = 1	*P* < 0.001 *P* < 0.001 *P* < 0.479
1,500 m		697 ± 34	712 ± 35^a^	717 ± 42^a^	722 ± 43^a^	718 ± 43^a^			
**3,000 m**		**687 ± 69**	**669 ± 42**	**668 ± 33**	**677 ± 39^b^**	**673 ± 32**			
1,500 m		674 ± 30	706 ± 29^a^	716 ± 36^a^	724 ± 41^a^	720 ± 42^a^	$R_{c}^{2}$ = 0.60 ICC = 0.50	(a) *F*_[4|4270]_ = 72 (b) *F*_[2|4077]_ = 35 (c) *F*_[8|4088]_ = 6	*P* < 0.001 *P* < 0.001 *P* < 0.001
3,000 m		673 ± 39	667 ± 33	674 ± 37	681 ± 41	687 ± 41^a^			
**5,000 m**		**709 ± 80**	**677 ± 43**	**677 ± 47**	**686 ± 44^b^**	**675 ± 38**			

Annual best times of the main race distance (dotted lines) were retrospectively extracted and compared to the individual swimmers’ annual best times of their secondary race distances. For the sake of clarity, significant differences to the previous age category are only indicated for the main race distance.

$R_{c}^{2}$, R-squared conditional; ICC, intra-class correlation coefficient.

Bold indicates main race distances.

Linear mixed model analysis: a) Main effect: age category (13–14 vs. 15–16 vs. 17–18 vs. 19–20 vs. 21–22 vs. 23+). b) Main effect: race distance (200 m vs. 400 m vs. 800 m vs. 1,500 m vs. 3,000 m vs. 5,000 m). c) Interaction effect: age category × performance level.

Bonferroni *post-hoc* comparison:

^a^ Significant difference to the main race distance.

^b^ Significant difference to previous age category.

## Discussion

The main findings of the present study are that swimmers' performances differentiate between world-class athletes and their lower ranked peers at an earlier age compared to that of runners. Due to the logarithmic development pattern, performance of swimmers plateaus earlier towards senior age compared to that of runners, which show a linear development pattern. Swimmers also show a greater within-sport distance variety and compete across all six race distances, whereas runners focus on only three to five race distances. While sprinters specialize most in both sports, within-sport variety of middle-distance swimmers covers more long-distance races rather than sprints. Additionally, (top-) elite swimmers specialize more than (highly-) trained swimmers.

### Performance development

Swimmers show a larger within-sport variety and compete in more events alongside their main race distance than runners, who specialize more and compete in fewer secondary race distances. As swimming technique is a dominant key performance indicator and important contributing factor to swimming performance, swimmers are able to compete over a greater variety of race distances within their specific swimming stroke than runners (14). The large contribution of swimming technique may also explain the logarithmic development pattern. Quick learning experience of technical elements may add to the physical development and allow for the steep performance curve at young age, which has also previously been shown in backstroke swimmers (47). In contrast, track runners primarily rely on physical fitness, and thus, develop linearly with later differentiation between performance levels (27, 29). The present findings are also supported by previous sprint data of track runners, which showed a similar linear development pattern after 10 years of age (48).

### Neuro-muscular aspects of sprint swimming

The present study shows that sprint swimmers specialize more than middle- and long-distance swimmers. Additionally, specialization affects performance level, as (top-) elite show earlier and greater specialization compared to (highly-) trained sprint swimmers. This higher degree in specialization may be due to the specific technical, metabolic and neuro-muscular demands that are required for world-class success (18, 19, 49). The neuro-muscular abilities have gained particular importance over the last decade, as they enhance in-water force production (50, 51) as well as start and turn performances, which have been shown to be distinguishing factors for swim races (52–55). As such, the push-off from a solid base during starts and turns allows swimmers to translate their full capacity of maximal strength and power into propulsion (20, 56). Therefore, the early introduction to dry-land training and strength-and-conditioning regimes is particularly important for sprint swimmers (21, 57) and may explain the earlier and higher degree of specialization in (top-) elite compared to (highly-) trained sprint swimmers. Furthermore, the scientific literature supports the early introduction of children and adolescents to strength training and shows that both sexes gain substantial strength even before puberty (58, 59). Additionally, establishing a solid technical foundation in regard to dry-land exercises and barbell lifting techniques during early stages of their swimming careers prepares sprinters for heavy lifts and minimizes injury risk at late junior and senior age (59, 60).

### Technical elements

In addition to the physiological and neuro-muscular specificities of sprint races, technical elements should be considered when discussing differences in specialization pattern. Swimming is traditionally considered an endurance sport, which requires high training volumes to maximize aerobic capacity and ingrain movement patterns specific to swimming technique (26). The relatively low density of water requires a progressively increasing hand velocity during the arm stroke, with minimal adjustments affecting optimal usage of the hydrodynamic lift, to allow for effective water-based movement (61, 62). Based on the fact that swimming involves different conversion of metabolic to mechanical power than land-based movement patterns, i.e., running (19), large training volumes are commonly prescribed to maximize the technical learning experience (23, 26). This aerobic culture of the sport may provide good development opportunities for middle-distance swimmers, whose distance variety covers more long-distance rather than sprint race. However, the higher velocities during sprint races require a higher cadence, which swimmers must translate into propulsion without losing traction, control of the water resistance and hydrodynamic lift during the in-water phase of the arm stroke (63–65). Additionally, the high cadence of sprint swimming does not allow for a completely relaxed arm and strict high elbow position during the overwater phase of the arm stroke. Instead, sprinter swing their arm forward with a more extended elbow and show fewer intra-cyclic gliding phases compared to middle- and long-distance swimmers (66, 67). This sprint-specific swimming technique, e.g., underwater hand trajectories, arm stroke, shoulder and hip roll at a high cadence (65, 68), are unlikely to develop during long-slow distance training. Instead, aerobic sessions should only be used to provide sufficient ability to recovery and to improve resilience to maximize volume of velocity-specific sets, i.e., 15–25 m bouts at and above race-pace (24, 69).

### Aerobic aspects and over-distance training

Throughout the development process, junior swimmers are typically developed from longer to shorter race distances, due to the over-distance oriented training approach (26, 70). In contrast, track sprinters (runners) aim for shorter sprints at younger ages and progress to longer race distances throughout adolescence in many countries, i.e., 60 m (U14), 80 m (U16) and 100 m [U18 and older (71, 72)]. While maximal sprint velocity and basic speed abilities can be developed from an early age in an athlete's career, insights from other endurance sports show that the aerobic capacity develops over consecutive years of training and that resilience for longer race distances increases with ages (73–75). Due to the technical challenge of translating a high stroke rate into propulsion (63–65), sprint swimmers in particular may benefit from an early focus on sprint speed, while their aerobic capacity could develop over the years of training (73, 75).

### Other disciplines in the sport

Training regimes are also affected by the other disciplines in the sport. Since most track and field disciplines rely on explosive strength, i.e., jumping, throwing and sprinting, the general athletic education is more oriented towards strength, speed and power. In contrast, with race times of more than one minute, the majority of swimming events rely mostly on aerobic energy contribution (33, 34), which explains the over-distance oriented training approaches (26). However, with the increasing importance of the acyclic elements, i.e., start and turn (52, 53, 55), placing more focus on dry-land training strategies from an early stage in swimming careers may help develop specific key performance indicators (21, 57). Future research should determine optimal ratios between dry-land and specific pool-based training, as well as volume and intensity during in-water sessions (21, 57, 76). This is also important in regard to the earlier performance plateaus evident in swimmers compared to runners, which indicates that key performance indicators related to swimming-specific physical fitness have not yet reached their full potential. Earlier and greater specialization on specific race distances and further development of strength and conditioning regimes may improve swimming performances and even world records in the future.

### Study limitations

The present study is based on race results that only allow retrospective analyses of performance pattern. By clustering performance groups, i.e., world-class finalists, international-, national- and regional-class athletes, in two sports, we aimed to discover the most promising development strategies, draw parallels and highlight differences between swimming and running. However, although development strategies of world-class athletes may have worked well in the past, they may not be the ideal for the future. Thus, the results of the present study can only provide a starting point for the development of new training strategies.

Infrastructural aspects need to be considered as well and may affect the development of optimal swimming technique (77). Compared to track runners, who have large track-and-field grounds and the option to complete aerobic sets, i.e., long-slow distance training, outside the running track, swimming pools are less spacious training facilities. In some countries, swimming clubs and teams have to share pools with the public and lane use is limited. Particularly at junior age, before swimmers train in national training centers (24, 70), sprint talents cannot be placed in a separate training group—due to infrastructure constraints—and often train in the same lane as middle-distance and long-distance swimmers. Hence, the volume-oriented programs do not allow for optimal training differentiation and development of sprint abilities. Even with knowledge of optimal development strategies, infrastructural aspects need to be considered and will affect knowledge transfer into the daily training and competition routine of coaches and athletes.

## Conclusion

Although, sprinters specialize more than middle- and long-distance swimmers, as do (top-) elite compared to (highly-) trained swimmers, the comparison to similar race distances (timewise) in track running indicates that swimmers have a larger within-sport distance variety compared to runners. The early performance plateau towards senior age, the large within-sport distance-variety and high reliance of key performance indicators on technical elements indicate that swimmers may not yet use their full physiological potential.

Since the majority of events favors aerobic and over-distance training, the landscape of competitive swimming does not provide an optimal environment for the development of sprint swimmers. The comparison with track running indicates that sprint swimmers may require an even earlier and higher degree of specialization with more attention to explosivity-oriented training regimes and frequent race-pace specific training sessions.

## Data Availability

The raw data supporting the conclusions of this article will be made available by the authors, without undue reservation.
